# 19-base pair deletion polymorphism of the dihydrofolate reductase (DHFR) gene: maternal risk of Down syndrome and folate metabolism

**DOI:** 10.1590/S1516-31802010000400008

**Published:** 2010-07-01

**Authors:** Cristiani Cortez Mendes, Joice Matos Biselli, Bruna Lancia Zampieri, Eny Maria Goloni-Bertollo, Marcos Nogueira Eberlin, Renato Haddad, Maria Francesca Riccio, Hélio Vannucchi, Valdemir Melechco Carvalho, Érika Cristina Pavarino-Bertelli

**Affiliations:** I MSc. Student, Genetics and Molecular Biology Research Unit, Faculdade de Medicina de São José do Rio Preto (Famerp), São José do Rio Preto, São Paulo, Brazil.; II PhD. Student, Genetics and Molecular Biology Research Unit, Faculdade de Medicina de São José do Rio Preto (Famerp), São José do Rio Preto, São Paulo, Brazil.; III PhD. Adjunct professor, Genetics and Molecular Biology Research Unit, Department of Molecular Biology, Faculdade de Medicina de São José do Rio Preto (Famerp), São José do Rio Preto, São Paulo, Brazil.; IV PhD. Titular professor, ThoMSon Mass Spectrometry Laboratory, Department of Organic Chemistry, Universidade Estadual de Campinas (Unicamp), Campinas, São Paulo, Brazil.; V PhD. Researcher at ThoMSon Mass Spectrometry Laboratory, Universidade Estadual de Campinas (Unicamp), Campinas, São Paulo, Brazil.; VI MSc. Student, ThoMSon Mass Spectrometry Laboratory, Universidade Estadual de Campinas (Unicamp), Campinas, São Paulo, Brazil.; VII PhD. Titular professor, Nutrition Laboratory, Department of Clinical Medicine, Universidade de São Paulo (USP), Ribeirão Preto, São Paulo, Brazil.; VIII PhD. Research associate, Centro de Medicina Diagnóstica Fleury, São Paulo, Brazil.

**Keywords:** Down syndrome, Polymorphism, genetic, Folic acid, Nondisjunction, genetic, Risk factors, Síndrome de Down, Polimorfismo genético, Ácido fólico, Não-disjunção genética, Fatores de risco

## Abstract

**CONTEXT AND OBJECTIVE:**

Polymorphisms in genes involved in folate metabolism may modulate the maternal risk of Down syndrome (DS). This study evaluated the influence of a 19-base pair (bp) deletion polymorphism in intron-1 of the *dihydrofolate reductase (DHFR)* gene on the maternal risk of DS, and investigated the association between this polymorphism and variations in the concentrations of serum folate and plasma homocysteine (Hcy) and plasma methylmalonic acid (MMA).

**DESIGN AND SETTING:**

Analytical cross-sectional study carried out at Faculdade de Medicina de São José do Rio Preto (Famerp).

**METHODS:**

105 mothers of individuals with free trisomy of chromosome 21, and 184 control mothers were evaluated. Molecular analysis on the polymorphism was performed using the polymerase chain reaction (PCR) through differences in the sizes of fragments. Folate was quantified by means of chemiluminescence, and Hcy and MMA by means of liquid chromatography and sequential mass spectrometry.

**RESULTS:**

There was no difference between the groups in relation to allele and genotype frequencies (P = 0.44; P = 0.69, respectively). The folate, Hcy and MMA concentrations did not differ significantly between the groups, in relation to genotypes (P > 0.05).

**CONCLUSIONS:**

The 19-bp deletion polymorphism of *DHFR* gene was not a maternal risk factor for DS and was not related to variations in the concentrations of serum folate and plasma Hcy and MMA in the study population.

## RESUMO

**CONTEXTO E OBJETIVO:**

Polimorfismos em genes do metabolismo do folato podem modular o risco materno para síndrome de Down (SD). Este estudo avaliou a influência do polimorfismo de deleção de 19 pares de base (pb) no íntron 1 do gene *dihidrofolato redutase (DHFR)* no risco materno para SD e investigou a associação entre esse polimorfismo e variações nas concentrações de folato sérico, homocisteína (Hcy) e ácido metilmalônico (MMA) plasmáticos.

**TIPO DE ESTUDO E LOCAL:**

Estudo transversal analítico realizado na Faculdade de Medicina de São José do Rio Preto (Famerp).

**MÉTODOS:**

105 mães de indivíduos com trissomia livre do cromossomo 21 e 184 mães controles foram avaliadas. A análise molecular do polimorfismo foi realizada pela reação em cadeia da polimerase (PCR) por diferença de tamanho dos fragmentos. O folato foi quantificado por quimioluminescência, e Hcy e MMA foram determinados por cromatografia líquida/espectrometria de massas sequencial.

**RESULTADOS:**

Não houve diferença entre os grupos em relação às frequências alélica e genotípica (P = 0,44; P = 0,69, respectivamente). As concentrações de folato, Hcy e MMA não mostraram diferença significativa entre os genótipos, entre grupos (P > 0,05).

**CONCLUSÕES:**

O polimorfismo de deleção de 19 pb do gene *DHFR* não é um fator de risco materno para SD e não está relacionado com variações nas concentrações de folato sérico, Hcy e MMA plasmáticos na população estudada.

## INTRODUCTION

Down syndrome (DS) is a genetic disease characterized, in most cases, by free trisomy of chromosome 21 caused by non-disjunction in maternal meiosis.^1,2^ James et al.^3^ were the first to observe an increased risk of chromosome non-disjunction due to abnormal folate metabolism, and this is responsible for abnormalities in the pattern of deoxyribonucleic acid (DNA) methylation.

Folate plays an essential role in several complex metabolic pathways, including those leading to DNA synthesis or conversion of homocysteine (Hcy) to methionine, which is then used to form the main DNA methylating agent: S-adenosyl methionine (SAM).^4^ Studies have shown that polymorphisms in genes encoding enzymes involved in this metabolic pathway, and higher concentrations of Hcy and lower folate concentrations, modulate the maternal risk factor for DS.^5-8^

The *dihydrofolate reductase* (*DHFR*) gene encodes an enzyme that catalyzes the conversion of dihydrofolate (DHF) into tetrahydrofolate (THF). It is also needed for the intracellular conversion of synthetic folic acid (which is consumed in supplements and fortified foods) to DHF and THF, which are the forms that participate in folate and Hcy metabolism.^9^ Johnson et al.^10^ described a 19-base pair (bp) deletion polymorphism in intron-1 of the *DHFR* gene and hypothesized that this polymorphism could be functional because the deletion removes a possible transcription factor binding site that affects gene regulation.

A study on the mothers of individuals with spina bifida showed that the messenger ribonucleic acid (mRNA) expression of the *DHFR* gene was 50% higher in the del/del genotype than in the ins/ins genotype.^11^ Moreover, in a study on women with breast cancer, Xu et al.^12^ also observed mRNA concentrations that were 2.4 and 4.8 times higher in individuals with the ins/del and del/del genotypes, respectively, in comparison with individuals with the ins/ins genotype.

This polymorphism has been correlated with modulation of the metabolites involved in the folate pathway. Gellekink et al.^13^ reported that the del/del genotype was associated with lower plasma Hcy concentrations, but did not find any association between this genotype and concentrations of serum and erythrocyte folate. Another study did not find any effect on Hcy concentration, but found increased plasma and erythrocyte folate levels in del/del individuals.^9^

To the best of our knowledge, no published studies have evaluated the association of 19-bp deletion polymorphism in intron-1 of the *DHFR* gene with the maternal risk of DS.

## OBJECTIVES

This study aimed to evaluate the influence of the 19-bp deletion polymorphism in intron-1 of the *DHFR* gene on the maternal risk of DS and to investigate the association between this polymorphism and variations in the concentrations of serum folate and plasma Hcy and methylmalonic acid (MMA), an indicator of vitamin B_12_ status.

## METHODS

This analytical cross-sectional study was carried out at Faculdade de Medicina de São José do Rio Preto (Famerp) and was composed of a case group formed by 105 mothers of DS children with karyotypically confirmed free trisomy 21 and a control group consisting of 184 mothers with healthy offspring and no experience of miscarriages. Informed consent was obtained from all these volunteers.

The mothers’ median ages at delivery (maternal age) and when the blood samples were obtained (age at presentation) were 30.4 years (range 12.9-46.3 years) and 43.1 years (range 22.5-69.3 years) in the case group, respectively, and 26.4 years (range 15.4-40.7 years) and 36.1 years (13.2-68.8 years) in the control group. The maternal age was calculated as the age of the mother at the birth of the DS child for the case group, and the age at the birth of the last child for the control group.

Fasting blood samples were collected for molecular and biochemical analysis (serum folate and plasma Hcy and MMA). DNA extraction was performed as previously described by Miller et al.^14^ and the 19-bp deletion polymorphism in *DHFR* gene was analyzed by means of the polymerase chain reaction (PCR) using the difference in the size of fragments to determinate the genotypes, using primer sequences described by Dulucq et al.^15^ The PCR-amplified fragments were analyzed by means of electrophoresis in 6% polyacrylamide gel.

Folate was quantified by means of chemiluminescence (Immulite Kit, DPC Medlab, Brazil) and liquid chromatography-tandem mass spectrometry was used to determine the plasma Hcy and MMA concentrations, as previously described.^16-18^

The existence of Hardy-Weinberg equilibrium was tested using the chi-square test, and genotype frequencies among the case and control mothers were compared by means of the likelihood ratio test and logistic regression. Mood’s median test was used to investigate the association between the polymorphism in the *DHFR* gene and variations in the serum folate and plasma Hcy and MMA concentrations. P values ≤ 0.05 were taken to be statistically significant.

## RESULTS

Table 1 presents the allele and genotype frequencies of 19-bp deletion polymorphism in the *DHFR* gene in the case and control groups. The allele frequencies were in Hardy-Weinberg equilibrium in both groups, and there were no differences in allele and genotype frequencies be­tween the DS and control mothers. There were no significant differences in the distributions of serum folate and plasma Hcy and MMA concentrations between the genotypes of the case and control groups (P > 0.05) (Table 2).

**Table 1.** Allele and genotype frequencies of 19-bp deletion polymorphism in intron-1 of the *dihydrofolate reductase (DHFR)* gene in the case and control groups of mothers

*DHFR*	Case mothers	Control mothers	P
Allele frequencies			
ins	0.50	0.46	0.44
del	0.50	0.54	
Genotype frequencies			
ins/ins	0.26	0.23	0.69
ins/del	0.48	0.46	
del/del	0.26	0.31	

**Table 2.** Distribution of serum folate, plasma homocysteine (Hcy) and plasma methylmalonic acid (MMA) concentrations according to genotypes of the 19-bp deletion polymorphism in intron-1 of the *dihydrofolate reductase (DHFR)* gene, in the case and control groups of mothers

Concentration	ins/ins	ins/del	del/del	P
**Case group**				
Folate (ng/ml)	14.10	11.85	12.30	0.57
Hcy (µmol/l)	6.21	7.25	5.34	0.20
MMA (µmol/l)	0.18	0.17	0.17	0.41
**Control group**				
Folate (ng/ml)	15.60	14.10	14.60	0.74
Hcy (µmol/l)	8.85	8.15	8.19	0.29
MMA (µmol/l)	0.15	0.14	0.14	0.98

## DISCUSSION

Independent of maternal age, DS has been associated with other etiological factors. Studies have shown that cell folate deficiencies result in aberrant DNA methylation, point mutations, chromosome breakage, defective chromosome recombination and aneuploidy of chromosome 21.^19-21^ Folate metabolism plays an important role in the synthesis of nucleotides and of SAM, which is the main donor of methyl groups for DNA, protein and phospholipid methylation reactions.^22^ Many genes are involved in these metabolic pathways and, in 1999, James et al.^3^ hypothesized that abnormalities in DNA methylation are a potential causative mechanism of meiotic non-disjunction. Their report stimulated considerable investigation into the possible role of folate metabolism in relation to the risk of having a DS child, and several studies have found this association.^5-8,23,24^

One important gene that is involved in this metabolism is *DHFR,* which encodes the enzyme responsible for reducing folic acid in THF.^9^ A common polymorphism in this gene, 19-bp deletion polymorphism in intron-1, has been correlated with modulation of the maternal risk of neural tube defects (NTDs),^10,11^ and of the concentrations of the metabolites involved in the folate pathway.^9,13,25^

Originally, Johnson et al.^10^ observed that the del/del genotype was related to increased risk of having offspring with spina bifida. On the other hand, another study has suggested that the del/del genotype has a protective effect and decreases the maternal risk of spina bifida,^11^ while yet another study reported no effect.^26^ Thus, the contribution of the 19-bp deletion polymorphism in intron-1 of the *DHFR* gene towards the risk of NTDs remains a matter of controversy.

Considering the high frequency of DS cases in families with higher risk of NTDs, and vice versa,^27^ and the fact that both diseases are influenced by the same genetic determinants of folate metabolism,^28^ it is possible that the 19-bp deletion polymorphism in intron-1 of the *DHFR* gene modulates the maternal risk of DS. However, in the present study, no association between this polymorphism and the maternal risk of DS was observed. In addition, no association was observed between the 19-bp deletion polymorphism of the *DHFR* gene and the folate, Hcy and MMA concentrations in the present study, although the presence of the polymorphic allele has been associated with increased concentrations of serum and erythrocyte folate, and reduction in the concentration of plasma Hcy in previous studies.^9,13,25^ The variations between studies may have been caused by nutritional and ethnic differences between the populations studied, as well as differences in sample sizes and other genetic factors.

One of the limitations of our study is that serum folate was quantified instead of erythrocyte folate. The erythrocyte folate content represents the time average of the folate concentrations occurring at the genesis of each red cell and is therefore less susceptible to rapid changes in diet.^29,30^ One study observed a significant difference in erythrocyte folate quantification between case and control mothers, which was not observed when quantifying serum folate.^31^ However, quantification of erythrocyte folate is more complex to perform^29.30^ and, for this reason, many studies have measured serum folate.^5,32-34^ In addition, a recent study has indicated that serum folate assays provide information that is equivalent to erythrocyte folate measurements for attempting to determine folate deficiency.^30^

## CONCLUSIONS

In this study, no evidence for an association between the 19-bp deletion polymorphism in intron-1 of the *DHFR* gene and the maternal risk of DS was observed. Moreover, this polymorphism was not related to variations in the concentrations of serum folate and plasma Hcy and MMA in the study population.
